# TRIXS: a multilayer grating solution towards highly efficient resonant inelastic tender X-ray scattering

**DOI:** 10.1038/s41377-025-02172-7

**Published:** 2026-01-21

**Authors:** Ke-Jin Zhou, Qiushi Huang, Mirian Garcia-Fernandez, Yeqi Zhuang, Stefano Agrestini, Shengyou Wen, Thomas Rice, Sahil Tippireddy, Jaewon Choi, Andrew Walters, Igor V. Kozhevnikov, Zhe Zhang, Runze Qi, Zhong Zhang, Hongchang Wang, Zhanshan Wang

**Affiliations:** 1 Diamond Light Source, Harwell Campus, Didcot, OX11 0DE United Kingdom; 2 Key Laboratory of Advanced Micro-Structured Materials, Ministry of Education, Institute of Precision Optical Engineering (IPOE), School of Physics Science and Engineering, Tongji University, Shanghai, 200092 China; 3 Shanghai Professional Technical Service Platform for Full-Spectrum and High-Performance Optical Thin Film Devices and Applications, Tongji University, Shanghai, 200092 China; 4 Shanghai Frontiers Science Center of Digital Optics, Tongji University, Shanghai, 200092 China; 5 Zhejiang Tongyue Optical Technology Co. Ltd, Huzhou, 313100 China; 6 Department of Materials Science and Engineering, Southern University of Science and Technology, Shenzhen, 518055 China; 7 Present Address: National Synchrotron Radiation Laboratory and School of Nuclear Science and Technology, University of Science and Technology of China, Hefei, 230026 China

**Keywords:** Optical spectroscopy, X-rays

## Abstract

Resonant inelastic X-ray scattering (RIXS) is a photon-in/photon-out spectroscopic technique which has become increasingly important for the condensed matter physics community. The development of the RIXS instrumentation in soft X-ray and hard X-ray range facilitated the research in 3*d* and 5*d* transition metal (TM)-based materials, respectively. However, the tender X-ray (2000–3000 eV) RIXS covering most of 4*d* TM-based materials severely falls behind due to the lack of high-performance energy dispersive optics. Here, we demonstrate the design and fabrication of a laterally graded multilayer grating (MLG) optics for the establishment of the tender RIXS at the I21 RIXS beamline in Diamond Light Source. The successful implementation of the MLG boosts the photon flux by more than an order of magnitude at the Sulfur *K*-edge (2475 eV) and the Ru *L*_*3*_-edge (2838 eV) in comparison to the solution of a single-layer coated grating (SLG). More importantly, MLG retains the high energy resolution of the SLG design (~10,000) and works continuously across the full range of 2000–3000 eV. It renders the I21 beamline as the very first RIXS facility in the world that covers both soft and tender X-rays (280–3000 eV) using a grating-based spectrometer for a wide range of science applications.

## Abstract (web summary)

Multilayer-grating-based spectrometer with high photon flux and moderate energy resolution for tender X-ray

## Graphical abstract

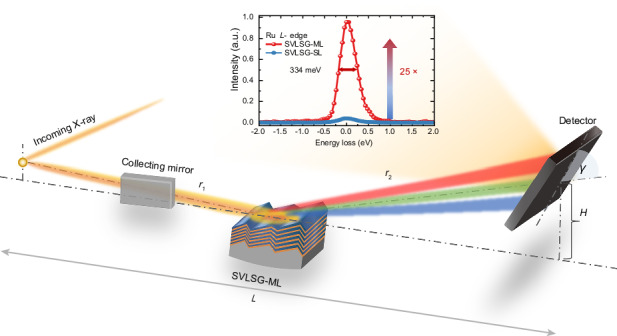

## Introduction

Over the last decades, resonant inelastic X-ray scattering (RIXS) made a paradigm shift for the fundamental research in material sciences, chemistry, and particularly the condensed matter physics^1–4^. RIXS is well established in probing the charge-neutral collective excitations including plasmons, magnons, orbitons, excitons, and phonons, as well as the ordering states, owing to its sensitivity to charge, spin, orbital, and lattice degrees of freedom. Observing and understanding the collective excitations is the cornerstone for the study of complex quantum materials. By working at core-level resonances, RIXS could provide complementary information about the electronic ordering or collective excitations of multiple magnetic ions in a material. Equipped with circular-polarized X-rays, RIXS can probe chiral phonons and possibly other chiral collective excitations^5^. For energy materials, RIXS has become a powerful tool in characterizing the principle of oxygen redox by providing the electronic structure of oxygen species^6–9^.

A large amount of strongly correlated quantum complex matter is formed based on 3*d* transition metals (TM) such as cuprate, iron pnictides, and nickelate superconductors^10–13^, Kagome Weyl semimetals showing anomalous quantum Hall effect^14^, two-dimensional van der Waals magnetic materials^15^. On the other hand, 5*d* TM-based materials exhibit a variety of unique properties like spin-liquid and spin-orbit coupled Mott insulating phases owing to the strong spin-orbit coupling and more spatially extended 5*d* orbitals. The advancement of research fields has benefited from the development of high-resolution RIXS instrumentations in both soft (400–1500 eV) and hard (4–15 keV) X-ray regions to access *L*-edges of 3*d* and 5*d* TM materials^16–22^.

**Fig. 1:**
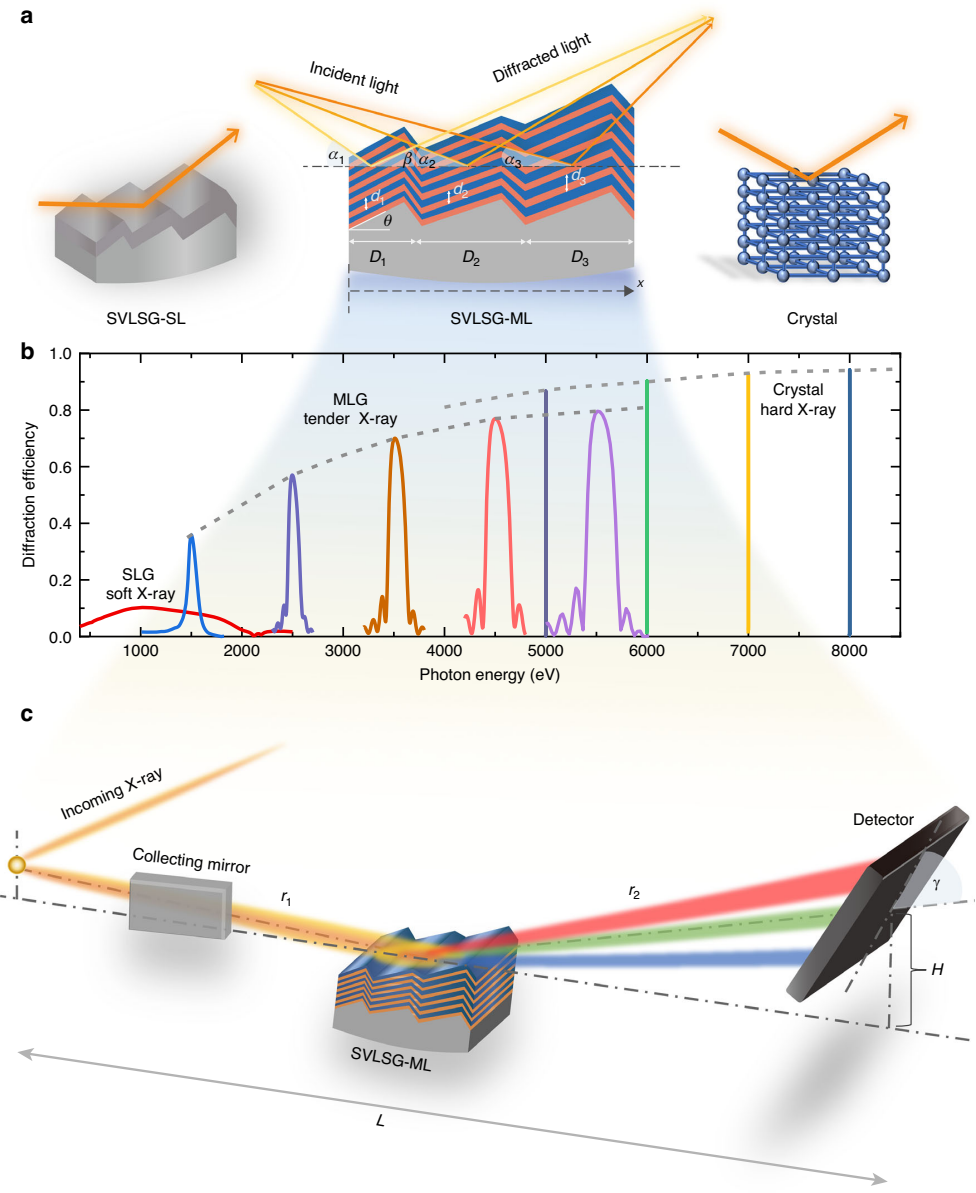
The structural and performance schematic diagram of SVLSG-ML applied to the spectrometer. **a** Illustrations of various dispersive optics - SLG, MLG, and crystal optics used for the soft, tender, and hard X-ray range, respectively. **b** Photon-energy-dependent diffraction efficiency of various dispersive optics. The SLG (1500 l mm^−1^ groove density, 0.68° blaze angle, and 2.72° anti-blaze angle) employs a 30-nm Pt coating under fixed 2° grazing incident angle. The MLG (identical groove density) incorporates Cr/C multilayers (*d*-spacing = 5.90 nm), with grazing angles optimized to 3.80°, 2.11°, 1.37°, 0.97°, and 0.71° for 1500, 2500, 3500, 4500, and 5500 eV, respectively. Si(111) crystal operates at 23.30°, 19.24°, and 16.41° grazing angles for 5000, 6000, and 7000 eV, respectively. **c** Schematic of the RIXS spectrometer based on the SVLSG-ML

Compared to 3*d* and 5*d* counterparts, 4*d* TM-based materials also manifest intriguing properties owing to the competition among the spin-orbit coupling, the Coulombic interaction, and the crystal-field splitting. For instance, α-RuCl_3_ is considered as a strong candidate for quantum spin liquid^23^, and Sr_2_RuO_4_ hosts unconventional superconductivity^24^. Two-dimensional van der Waals systems NbS_2_ exhibit superconductivity and charge density waves, while MoX_2_ (X = S, Se, Te) are semiconductors promising for the spintronic application^25,26^. Unfortunately, the RIXS instrumentation at the tender energy range (2–4 keV) covering *L*-edges of most 4*d* TMs lags significantly behind, primarily due to the lack of suitable dispersive optics. It is known that conventional single-layer coated gratings (SLGs) and crystal optics work well for the soft X-ray and hard X-ray regions respectively^16–22,27^(Fig. 1a). Nevertheless, the diffraction efficiency of high line density SLG drops to below 5% at the tender X-ray range. Using (10-2) oriented quartz crystals, an intermediate X-ray RIXS (IRIXS) spectrometer was constructed successfully for Ru *L*-edge (~2838 eV), achieving a total energy resolution of 100 meV at PETRA III^28^. However, IRIXS and similar spectrometers are not easily adaptable for other 4*d* TM materials due to narrow Darwin width of natural crystals^29^ (Fig. 1a, b).

Multilayer gratings (MLG) can overcome the limitations of both SLG and crystal analyzer optics. By fulfilling simultaneously the generalized-Bragg diffraction of the multilayer and the grating equation, a maximum efficiency up to 60% was achieved at 3–4 keV, which is more than one order of magnitude higher than a typical SLG^30–32^. Moreover, it can continuously cover the tender X-ray range and be extendable to hard X-rays. MLGs have been implemented at the beamlines in SOLEIL and BESSY-II^33–36^. In these beamlines, lateral uniform multilayers are coated on constant-line-spacing and plane-shaped gratings in monochromators. To apply MLG for the grating-based spectrometer as a solution of the tender X-ray RIXS (TRIXS), one cannot simply use uniform *d*-spacing multilayers, as they are incompatible with the typical optics design of spectrometers with spherical variable-line-spacing gratings (SVLSGs) covering a large acceptance angle^16–18^.

In this paper, we demonstrate for the first time the design and fabrication of a new laterally graded MLG with spherical variable-line-spacing (SVLS) structure for the TRIXS (2000–3000 eV) at I21 beamline in Diamond Light Source. The MLG design accommodates a large beam divergence from the sample while maintaining the high resolution and high efficiency. Remarkably, the MLG solution boosts the photon flux of the RIXS spectrometer by maximal 25 times in comparison to the conventional SLG. Moreover, the MLG does not compromise the high energy resolution of SLG design (~10,000) and covers continuously 2000–3000 eV by employing three multilayer coating stripes across the transverse direction of the grating. We believe the work opens a new avenue for RIXS studies at the tender X-ray range on systems with various forms, such as small crystals, thin films, and heterostructures. The successful implementation of the MLG makes the I21 the only RIXS facility in the world that covers both soft and tender X-rays (280–3000 eV) using a grating-based spectrometer.

## Results

### Design of the MLGs for TRIXS spectrometer

We consider the combination of multilayer coating and a blazed SVLS grating for the grating-based RIXS spectrometer at I21 beamline to achieve the highest possible photon flux at the tender X-ray range of 2000–3000 eV. To realize this, the grating equation (Eq. (1)) and the generalized-Bragg condition (Eq. (2)) of the multilayer must be satisfied simultaneously, so that the diffracted wave from all multilayer interfaces interferes constructively in the grating diffraction direction.

1 $$D(\cos \,\beta -\,\cos \,\alpha )=n\lambda$$

2 $$\sin \,\beta +\,\sin \,\alpha \approx \frac{\lambda \,\cos \,\theta }{d}$$

Here, *α* and *β* are the grazing incident angle and diffraction angle, respectively. *n* is the diffraction order, *θ* is the blaze angle of the grating, *D* is the grating period, *d* is the multilayer *d*-spacing, and *λ* is the X-ray wavelength. A more accurate form of the generalized Bragg condition, including the refraction, absorption, and the effect of groove shape, can be seen in ref. ^37^. Considering the small grazing incident angle, the following relationship (Eq. (3)) can be deduced

3 $$\alpha \approx \frac{\lambda \,\cos \,\theta }{2d}+\frac{nd}{D\cdot \,\cos \,\theta }$$

Thus, to achieve the maximum diffraction efficiency, the grazing incident angle, grating period, and multilayer *d*-spacing need to be matched at each photon energy. This brings significant complexity to the design of the spectrometer using MLG.

The schematic layout of the spherical variable line spacing grating (SVLSG) RIXS spectrometer at I21 beamline is shown in Fig. 1c. The single-layer metal coated grating (SVLSG-SL) has a cylindrical shape with a total length of 200 mm and a radius of curvature of *R* = 115.72 m in the longitudinal (beam propagation) direction. Although the outgoing X-rays are collimated in the horizontal direction by the collecting mirror, they are divergent in the vertical direction, resulting in a wide range of incident angles (*α*_1_–*α*_3_) over the length of the grating. It is critical for the multilayer coated SVLSG (SVLSG-ML) to match the angular span of the incident angle and the variable grating period to achieve the high diffraction efficiency. For this, we designed the laterally graded MLG to work at the tender X-ray energy of 2000–3000 eV. The detailed parameters of the SVLSG used in I21 are listed in Table 1. We show below the systematic study and optimization of the SVLSG-ML.

**Table 1:** Parameters of the SVLSG-ML structure

Multilayer grating		Structure parameters	
Blaze angle (°)		0.68	
Apex angle (°)		176.6	
*a*_0_ (l mm^−1^)		1500.017	
*a*_1_ (l mm^−2^)		−4.522 × 10^−2^	
*a*_2_ (l mm^−3^)		−4.123 × 10^−4^	
*a*_3_ (l mm^−4^)		3.4 × 10^−7^	
Tangential radius (mm)		115,720	
Tangential slope error (μrad)		≤0.1	
Multilayer materials		Cr/C	
Multilayer *d*-spacing (nm)	SVLSG-ML-1 8.36–8.84	SVLSG-ML-2 7.29–7.65	SVLSG-ML-3 5.73–5.90
Effective length (mm)		180	
Strip width (mm)		9	

**Fig. 2:**
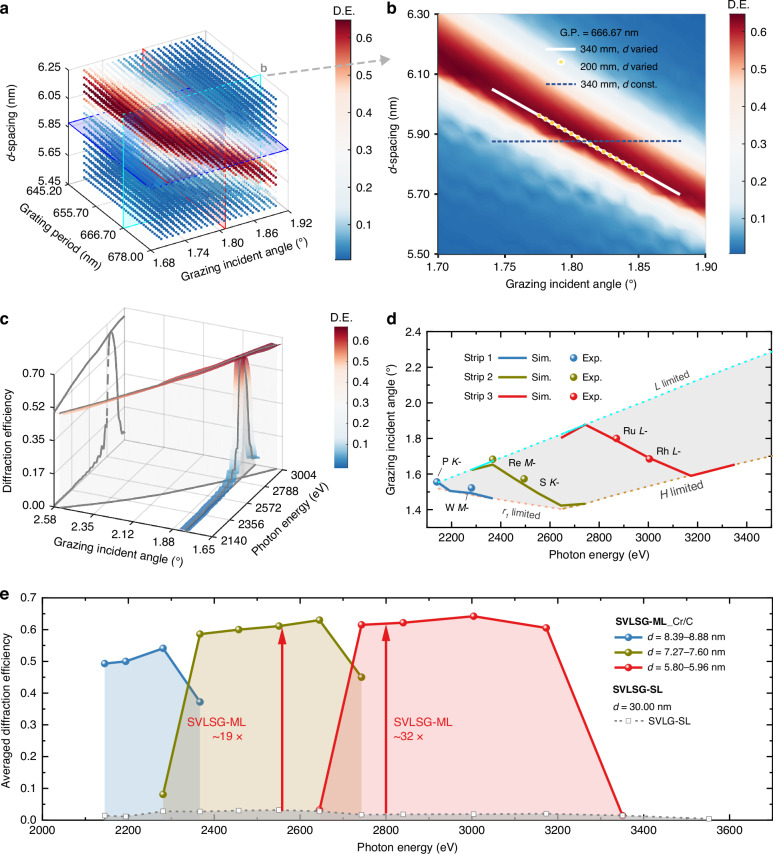
The parameter optimization of SVLSG-ML. **a** 3D visualization^43^ of the optimized diffraction efficiency of the SVLSG-ML at 2838 eV as a function of incident angle, *d*-spacing, and grating period. **b** Diffraction efficiency dependence on grazing incident angle and *d*-spacing for a grating period of 666.67 nm. The white solid curves and yellow dots in (**b**) represent SVLSG with a variable *d*-spacing for a grating length of 340 mm and 200 mm, respectively. Blue dashed curves denote SVLSGs with a constant *d*-spacing (grating length = 340 mm) for comparison. **c** The diffraction efficiency dependence of SVLSG-ML on the photon energy and the incident angle. **d** Variation of the incident angle as a function of photon energy, where the gray area denotes the effective working zone limited by the spectrometer’s mechanical constraints. The solid lines show the optimum theoretical angles of the SVLSG-ML, and the scatter points represent the measured angles with maximum efficiency. **e** The average theoretical diffraction efficiency varied with photon energy, with solid lines for SVLSG-ML and dashed lines for SVLSG-SL

The SVLSG-ML structure was first optimized at one of the most interesting photon energy values, the Ru *L*_3_-edge (2838 eV). Cr/C multilayer was selected, given its high reflectance in the whole tender X-ray range. The grating period at the center position of SVLSG is 666.67 nm (1500 l mm^−1^) with a blaze angle of 0.68° and a grazing incident angle of 1.81°. The overall relationship of the *d, D*, *α*, and their combined impact on the efficiency are simulated using coupled-wave theory, and the results are shown in Fig. 2a. Note that the incident angle has been corrected by considering the radius of curvature of the cylindrical substrate. The variation of the optimal *d*-spacing with grating period is relatively small. Furthermore, for the SVLSG used at I21, the grating period only changes from 666.13 nm to 669.46 nm, and the *d*-spacing can remain almost constant. The variation of the optimal *d*-spacing with incident angle is much larger than for conventional SLGs, constrained by the narrow bandwidth of multilayer. Figure 2b shows the relationship between the *d*-spacing and the incident angle. In this diffraction geometry, the full width half maximum (FWHM) of the angular bandwidth of incident angle is only around 0.16°. For a constant *d*-spacing multilayer design, using a 200 mm long SVLSG-ML, the diffraction efficiency drops from ~65% in the center to ~28% near the edge (Fig. 2b). Further increasing the grating length to 340 mm will reduce the diffraction efficiency to around 10% at the edge (dashed line in Fig. 2b). To overcome the issue, we made the design and changed the constant *d*-spacing multilayer to a laterally graded one with variable *d*-spacings. The gradient can be optimized to exactly match the different incident angles to satisfy the generalized Bragg conditions as indicated by the solid line in Fig. 2b. Thus, a high efficiency of ~60% at 2838 eV can be realized over the entire grating (Fig. 2b).

A key difference compared to conventional SLGs is that MLGs have a relatively narrower bandwidth, requiring the incident angle to be tuned at different energies to fulfill the generalized Bragg condition. The SVLSG-ML, designed for 2838 eV photon energy, has an effective FWHM energy bandwidth of about ~120 eV under a fixed incident angle (Fig. 2c). By varying the incident angle, the same SVLSG-ML can, in principle, achieve a comparable diffraction efficiency at other energy positions. As shown in Fig. 2c, the entire tender X-ray energy range of 2000–3000 eV can be covered with a high efficiency of ~50% when the grazing incident angle varies from 2.55° to 1.68°.

In addition, to fulfill the grating diffraction and Bragg conditions, the optics design needs to obey the mechanical limitations of the spectrometer. For a RIXS spectrometer, the lower limit of the object distance *r*_1_, the lower limit of the detector height *H*, and the upper limit of the detector length *L* constrain the incident angular range for each energy position (marked as gray area in Fig. 2d). Considering the mechanical constraints, the SVLSG-ML with a single set of lateral gradient *d*-spacing cannot work for the whole tender X-ray range. For instance, the above-designed SVLSG-ML (stripe 3) can only cover 2743 eV (Au *M*_3_-) to 3173 eV (Pd *L*_3_-), beyond which the diffraction efficiency reduces immediately to < 10% due to the mismatched incident angle (Fig. 2e). To cover the entire energy range of 2000–3000 eV, we designed two extra laterally graded multilayers. Detailed parameters are shown in Table 1. The corresponding incident angles and working energies of the three stripes are shown in Fig. 2d. Without violating the mechanical constraints, stripe 1 covers 2145 eV (P *K*-) to 2281 eV (W *M*_3_-), and stripe 2 can reach the energy range from 2367 eV (Re *M*_3_-) to 2645 eV (Pt *M*_3_-). The average diffraction efficiency of the three SVLSG-MLs can achieve > 50% (Fig. 2e). In comparison, the original blazed SVLS grating is coated with a 30 nm thick Pt single layer (SVLSG-SL) whose diffraction efficiency is less than 3% from 2000 to 3000 eV (Fig. 2e). By coating with multilayers, the theoretical efficiency can be boosted by 19–32 folds. A more detailed theoretical simulation of the SVLSG-ML over different positions of the grating and their spectral response is shown in Supplementary Fig. S1 in the supplementary information.

The diffraction geometry of a MLG still obeys the grating equation^38^. It means that the diffraction light paths and aberration correction effects of the SVLSG-ML follow that of the SVLSG-SL. The theoretical energy resolution of SVLSG-ML should retain what can be achieved by SVLSG-SL under the same incident angles.

### Fabrication of the MLG

**Fig. 3:**
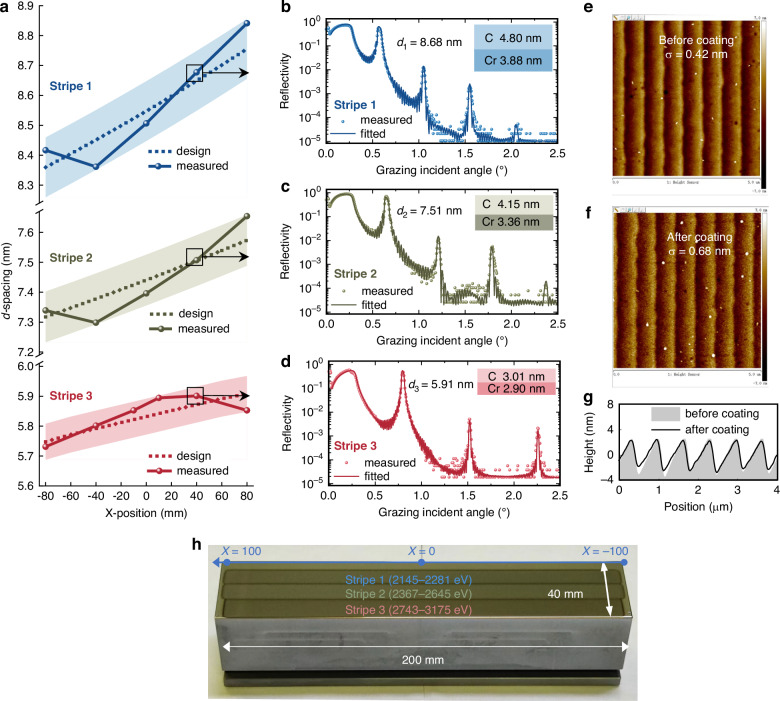
Structural characterization of fabricated SVLSG-ML. **a** The designed (dotted line) and measured (solid line) *d*-spacing distribution of three stripes. The color filling part represents the theoretical tolerance area. The GIXRR measured (dots) and fitting (solid line) curves of the reference multilayer sample at the position X = 40 mm of stripe 1 (**b**), 2 (**c**), and 3 (**d**) with the fitted thickness of the Cr and C layers. AFM images of grating before (**e**) and after (**f**) coating stripe3. **g** The grating profile curves obtained from the AFM images. **h** The photo of the grating coated with three stripes

Three Cr/C laterally graded multilayer stripes were deposited directly on the Pt single layer coated grating using the magnetron sputtering technique^39^. The designed and measured *d*-spacing of the multilayer reference samples are presented in Fig. 3a, which indicates that the deposited thickness variation is within the designed error bar (±100 pm for strip 1, ±85 pm for strip 2, ±65 pm for strip 3). The grazing-incident X-ray reflectivity (GIXRR) measurements and fitted results of three stripes multilayers are shown in Fig. 3b–d. The interface widths of the multilayers are 0.6 nm, indicating relatively sharp interfaces of the laterally graded multilayer. The surface morphology of the grating before (Fig. 3e) and after (Fig. 3f) coating was measured by the atomic force microscopy (AFM). The groove profile of the blazed grating is well replicated through the multilayer coating. A slight increase of the surface roughness of the facets from 0.42 nm to 0.68 nm was found after coating. This can be further improved if a smoother grating surface was used as the substrate. The high-quality multilayer and conformal layer growth are essential for the performance of the SVLSG-ML. A photo of the SVLSG-ML is shown in Fig. 3h, and each stripe is about 9 mm wide.

### Tender X-ray measurements

**Fig. 4:**
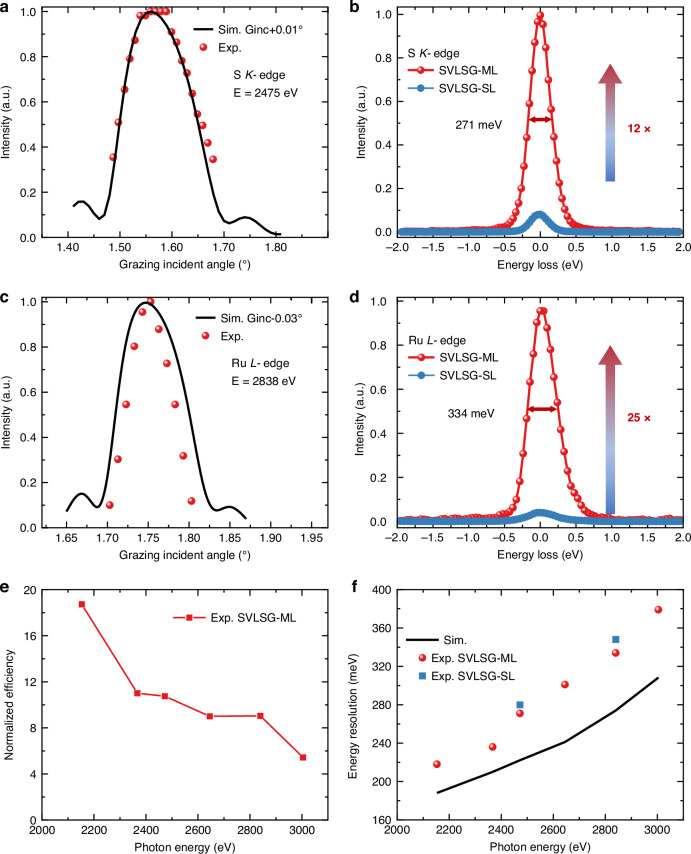
Tender X-ray performance characterization. The measured (dots) and simulated (solid line) rocking curves at 2475 eV (**a**) and 2838 eV (**c**). Instrumental energy resolution of SVLSG-ML (red) and SVLSG-SL (blue) at 2475 eV (**b**) and 2838 eV (**d**). **e** Normalized efficiency of the SVLSG-ML spectrometer as a function of the photon energy. **f** Measured instrumental energy resolution (red dots for SVLSG-ML and blue dots for SVLSG-SL) and simulated energy resolution (solid line)

The diffraction intensity versus the incident angle, *i.e*., the rocking curve, was measured at 2475 eV (S *K*-edge) (Fig. 4a) and 2838 eV (Ru *L*_3_-edge) (Fig. 4c). The optimal working incident angles of typical energies are further shown in Fig. 2d. The experimental results are consistent with the theoretical calculations suggesting the high quality of the fabricated MLG and the validity of the theory.

The total instrumental energy resolution of the SVLSG-ML and SVLSG-SL at 2475 eV and 2838 eV was obtained by measuring the non-resonant elastic scattering from an amorphous carbon sample (Fig. 4b, d). Strocov et al. identified an optimal operation mode of a RIXS spectrometer where a maximal grating illumination (i.e., the maximal acceptance) can be achieved through combined adjustment of the spectrometer parameters in which coma and higher-order aberrations are minimized^40^. Although the grazing incident angle is constrained in the current multilayer design, it has a very small angular difference of 0.23° across the full energy range (Fig. 2d). To maintain a good total energy resolution, we optimized the maximal illumination of the grating to be 160 mm corresponding to a vertical acceptance angle of 2.6 mrad which is smaller than the ideal maximal acceptance. Compared to the full usage of the entire lateral length of 190 mm, the photon flux is reduced by about 16%. However, we retain the best possible energy resolution for the major tender X-ray range.

Concerning the gain of the photon flux, the integrated intensity of the elastic peak from the SVLSG-ML is 12 times higher than that of SVLSG-SL at 2475 eV and 25 times higher at 2838 eV. The results are close to the theoretical predictions of 19 times and 32 times, respectively (Fig. 2e). The small discrepancy may be due to the interfacial imperfection and slight deviations of the laterally graded thickness distribution of the multilayer.

The photon efficiency is normalized to the incoming photon flux at each energy (Fig. 4e). Note that the deviation of the photon efficiency trend of the experimental data compared to the theoretical prediction (Fig. 2e) may be due to the energy-dependent scattering efficiency of the carbon sample and the quantum efficiency of the area detector. The latter two are unknown in the tender X-ray range and thus were not considered for the normalization. The total instrumental energy resolution (FWHM) of the SVLSG-ML and SVLSG-SL at various energies is shown in Fig. 4f. The energy resolution of the SVLSG-SL was only measured at two energy values, 2475 eV and 2838 eV. The total energy resolutions are maintained after the multilayer deposition and align well with the theoretical prediction. The overall total resolving power using the SVLSG-ML is close to ~10,000 across the entire 2000–3000 eV range. Note that the beamline energy resolution was not experimentally determined, it was estimated to be comparable to that of the spectrometer under the exit slit opening of 20 μm. It is evident that the MLG can provide more than an order of magnitude gain of the photon flux while maintaining the energy resolution, which is crucial for photon-hungry RIXS technique.

**Fig. 5:**
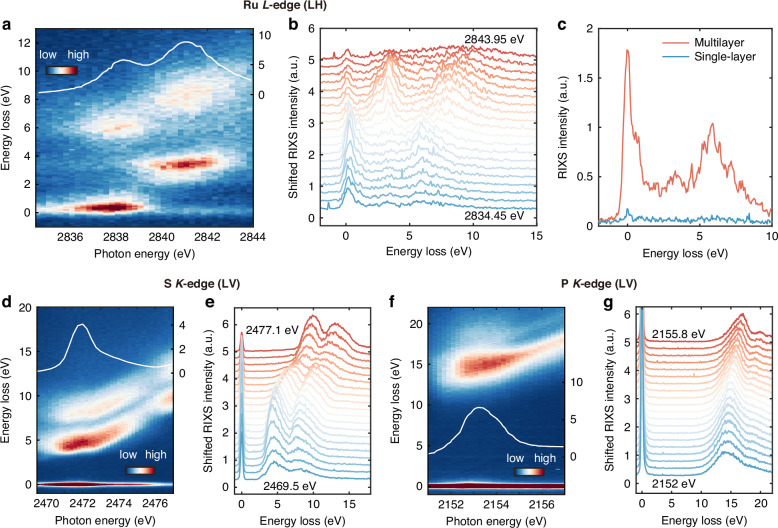
Energy-dependent RIXS spectra measured by SVLSG-ML and SVLSG-SL. **a**, **b** The incident energy-dependent RIXS spectra collected from Ca_2_RuO_4_ single crystal sample at Ru *L*_3_-edge using SVLSG-ML. **c** The RIXS spectra at the Ru *L*_3_-edge collected under SVLSG-ML and SVLSG-SL. The incident angle is fixed at 20°. The total energy resolution (FWHM) was 334 meV. The beamline exit slit opening is 20 μm corresponding to a photon flux of about 2 × 10^11^ phs s^−1^ and an estimated beamline energy resolution of 220 meV. **d**, **e** The incident energy-dependent RIXS spectra collected from Sulfur powder sample at S *K*-edge using SVLSG-ML. The incident angle is fixed at 20° with an exit slit opening at 20 μm. The total FWHM energy resolution was 266 meV. **f**, **g** The incident energy-dependent RIXS spectra collected from H_2_KOP powder sample at P *K*-edge using SVLSG-ML. The incident angle is fixed at 20° with an exit slit opening at 20 μm. The total FWHM energy resolution was 220 meV

We now demonstrate the excellent performance of the spectrometer based on SVLSG-ML by acquiring representative RIXS spectra from several reference samples. Figure 5a, b shows the incident energy-dependent RIXS spectra of Ca_2_RuO_4_ single crystal sample across the Ru *L*_3_-edge using π linear polarization. The resonances *E*_1_ at ~2838 eV and *E*_2_ ~ 2841 eV correspond to 2*p*_3/2_ −> 4*d t*_2g_ and 2*p*_3/2_ −> 4*d e*_g_ transitions in the Ru *L*_3_- X-ray absorption spectrum (XAS), respectively. The peak A and B is located at energy loss ~0.3 eV and ~3.2 eV resulting from the intra-*t*_2g_ and *t*_2g_ −> *e*_g_ *dd* excitations, respectively. The peak C at energy loss of ~ 6 eV and D ~ 8 eV originate from the charge-transfer excitations. The overall energy-dependent RIXS spectra are highly consistent to the published data^41^. Figure 5c shows the efficiency enhancement of a single RIXS spectrum collected by the SVLSG-ML compared to the SVLSG-SL. Note that the acquisition time of each spectrum is only three minutes. The spectrum collected under SVLSG-SL is near the detection limit, demonstrating the impractical use of SVLSG-SL for the TRIXS. The implementation of the SVLSG-ML makes the TRIXS possible for complex experiments such as fine temperature-dependence, detailed momentum-dependence, strain-tuning studies, and thin film samples.

Besides Ca_2_RuO_4_ as a representation of complex 4*d* quantum materials, we also tested reference samples relevant for the field of energy materials. Figure 5d, e shows the incident energy-dependent RIXS spectra collected at the S *K*-edge from pure Sulfur powder sample. Below the S *K*- absorption edge (~2472 eV), two Raman-like features at ~4 eV and ~8 eV are possibly charge-transfer excitations. Above the absorption edge, the two excitations smoothly merge into the two fluorescence-like excitations in the diagonal direction. Figure 5f, g displays the incident energy-dependent RIXS spectra collected at the P *K*-edge from H_2_KOP powder sample. Similarly, below the absorption edge (~2154 eV), two Raman-like excitations appear at ~15 eV and ~18 eV, much higher than that of S *K*-RIXS, which may suggest a relatively larger charge-transfer energy. The excitations merge into fluorescence-like excitations above the absorption edge. Again, it is promising to obtain high statistical data within a short acquisition time (six minutes) owing to the enhanced efficiency of the SVLSG-ML. Future experiments on energy materials under various conditions are possible, making the RIXS technique not only relevant for the complex quantum materials but also useful for the study of energy materials.

## Discussion

In our work, a highly efficient tender X-ray dispersive optics is designed and fabricated using a SVLS blazed grating combined with laterally graded multilayers. The optics has been successfully used in the I21-RIXS spectrometer at Diamond Light Source. Compared to the conventional single-layer Pt coated grating, the multilayers boost the diffraction efficiency by 12–25 folds across the tender X-ray range (2000–3000 eV). The significant improvement in photon throughput is at zero cost of the total energy resolution. Test RIXS measurements on quantum materials and energy materials show rich profiles of various excitations within several minutes’ acquisition time. The implementation makes the I21 across both soft and tender X-ray regions the very first RIXS facility using the grating optics. A comparison of the performance of different RIXS instruments is shown in Supplementary Table S1 in the supplementary information.

Currently at the beamline monochromator only a conventional SLG is used, which has an averaged diffraction efficiency of ~5%. It is noticeable that a further gain in photon flux can be made if a MLG can be implemented. In terms of the working energy range, the Cr/C MLG itself can work up to ~6 keV with efficiency >50% ^30^. By changing the material to Ni/C, MLGs can be further extended up to ~8 keV. Using W/B_4_C, they can work towards the soft X-ray of 1 keV. The potential extension of the MLGs towards both the low and high X-ray energy range would significantly boost the science applications in condensed matter physics and energy materials covering the *M*- edges of rare-earths, all the *L-*(*M-*) edges of 4*d* (5*d*) transition metals, and the *K*- edges of some 3*d* transition metals elements. Note that for the soft X-ray range, the photon flux enhancement will be smaller compared to the tender and hard X-ray counterparts.

TRIXS spectrometer features a moderate resolving power of ~10,000. For the studies of low-energy excitations in many quantum materials, a higher resolving power would be desirable. There are two possible routes to improve the resolving power, i.e., increasing the grating line density or working at higher diffraction orders^38^. For instance, by working at the 3^rd^ diffraction order, one could obtain an energy resolution of the spectrometer ~82 meV at 2500 eV by a small loss of the diffraction efficiency of 10%. The improvement of the resolving power does not add extra requirement on the X-ray beam parameters such as the beam divergence, the source size, and the beam stability. Note that a similar MLG is required for the beamline to achieve a higher total energy resolving power. In the future, TRIXS can have a similar or better energy resolving power compared to IRIXS spectrometer using natural crystal analyzers. More importantly, TRIXS continuously covers a very large incident energy range (>1 keV) without the need to exchange dispersive optics. Also, RIXS spectra up to tens of eV energy emission window can be obtained in a single exposure without scanning the spectrometer, which can boost significantly the experimental efficiency. It is noteworthy that the MLG is not only useful for RIXS technique but also widely applicable to many other X-ray spectroscopic techniques, such as tender X-ray photoemission spectroscopy or X-ray scattering techniques.

## Methods

### Design and fabrication of the SVLS-ML

The diffraction efficiency of SVLSG-ML was calculated using the RETICOLO^42^ software package, which is based on the rigorous coupled-wave analysis (RCWA) method. The laterally graded MLs were deposited using a linear magnetron sputtering system. The grating was moving linearly across the sputtering area with a designed speed profile so as to control the deposited thickness gradient. After deposition, the reference ML samples were characterized using grazing incidence X-ray reflectometry (GIXRR) measurement with a Bruker D8 DISCOVER instrument. The surface roughness and groove profile of the grating before and after coating were characterized using AFM on a Bruker Dimension Icon system with a scan area of 5 µm × 5 µm.

### X-ray measurement

X-ray measurements were performed at I21 beamline at Diamond Light Source^18^. The carbon sample was glued onto a copper sample holder and fixed at an incident angle of 80°. The spectrometer scattering angle was fixed to 154°. The beamline exit slit was fixed to 20 μm throughout the measurement, for which the analytical calculations suggest that incident X-rays have comparable energy resolution as that of the spectrometer. The incoming photon flux using the dedicated tender X-ray beamline grating VPG4 is about 2 × 10^11^ phs s^−1^ covering most of the energy range (2200–3000 eV). The total energy resolution and the photon efficiency were measured based on the non-resonant elastic scattering from the carbon sample.

## Supplementary information

Supplementary information for TRIXS: A multilayer grating solution towards highly efficient resonant inelastic tender X-ray scattering

## Data Availability

The data that support the findings of this study are available from the corresponding authors upon reasonable request.
