# Supplementary material for: TRIXS: a multilayer grating solution towards highly efficient resonant inelastic tender X-ray scattering
Source: Light Sci Appl. 2026 Jan 21;15:76. doi: 10.1038/s41377-025-02172-7 (PMC12820124; doi:10.1038/s41377-025-02172-7)
Supplement: Supplementary file 1 — Supplementary information for TRIXS: A multilayer grating solution towards highly efficient resonant inelastic tender X-ray scattering [file 41377_2025_2172_MOESM1_ESM.docx]

**Supplementary information for**

**TRIXS: A multilayer grating solution towards highly efficient resonant inelastic tender X-ray scattering**

Ke-Jin Zhou^1,7*^, Qiushi Huang^2,3,4,5*^, Mirian Garcia-Fernandez^1^, Yeqi Zhuang^2,3,4,6^, Stefano Agrestini^1^, Shengyou Wen^2,3,4^, Thomas Rice^1^, Sahil Tippireddy^1^, Jaewon Choi^1^, Andrew Walters^1^, Igor V. Kozhevnikov ^2,3,4^, Zhe Zhang^2,3,4^, Runze Qi^2,3,4^, Zhong Zhang^2,3,4^, Hongchang Wang^1*^, Zhanshan Wang^2,3,4*^

^1^ Diamond Light Source, Harwell Campus, Didcot, OX11 0DE, United Kingdom

^2^ Key Laboratory of Advanced Micro-Structured Materials, Ministry of Education, Institute of Precision Optical Engineering (IPOE), School of Physics Science and Engineering, Tongji University, Shanghai 200092, China

^3^ Shanghai Professional Technical Service Platform for Full-Spectrum and High-Performance Optical Thin Film Devices and Applications, Tongji University, Shanghai 200092, China

^4^ Shanghai Frontiers Science Center of Digital Optics, Tongji University, Shanghai 200092, China

^5^ Zhejiang Tongyue Optical Technology Co., Ltd. Huzhou 313100, China.

^6^ Department of Materials Science and Engineering, Southern University of Science and Technology, Shenzhen, 518055, China.

^7^ Present address: ﻿National Synchrotron Radiation Laboratory and School of Nuclear Science and Technology, University of Science and Technology of China, Hefei 230026, China

Corresponding authors:

^*^ Email: kjzhou@ustc.edu.cn

^*^ Email: huangqs@tongji.edu.cn

^*^ Email: hongchang.wang@diamond.ac.uk

^*^ Email: wangzs@tongji.edu.cn

Figure S1

Table S1

**1.1 Design and theoretical performance of the SVLSG-ML**

The design of the resonant inelastic X-ray scattering (RIXS) spectrometer geometry determines the grazing incident angle and line density distribution over the spherical variable line spacing grating (SVLSG), which requires the lateral-graded multilayer (ML) along the tangential direction of the grating surface. The *d*-spacing of the multilayer at different positions on the SVLSG was optimized to achieve the maximum diffraction efficiency at the designed energies. Considering the variation of the incident angles is similar over the energy band, a single graded ML can meet the requirements at different energies. Fig. S1a displays the designed SVLSG-ML parameters (stripe 3) and the diffraction efficiency at 2838 eV. The grating line density varies from 1501.21 l mm^-1^ to 1493.93 l mm^-1^, while the incident angle changes from 1.844° to 1.782°. The optimized *d*-spacing ranges from 5.80 nm to 5.96 nm. As a result, a uniform diffraction efficiency of 0.64 is achieved across the entire stripe 3.

To evaluate the spectral response of the SVLSG-ML, simulations of the diffraction efficiency versus energy were performed for different local positions along the grating, as shown in Fig. S1b. The results indicate nearly identical response profiles across the grating, with a full width at half maximum (FWHM) of 114 eV for all curves. These local response curves were averaged to represent the overall spectral performance of the SVLSG-ML. The averaged spectral response was also computed for photon energies of 2145 eV, 2281 eV, 2457 eV, 2645 eV, 2838 eV, and 3004 eV, which are indicated by dashed lines in Fig. S1c. Due to the mechanical constraints and the required working angle of the spectrometer, the SVLSG-ML cannot always work at its peak efficiency condition, as seen in the slight deviation between the designed efficiency (solid dot) and the peak efficiency of the grating. The theoretical efficiency of SVLSG-SL coated by a 30 nm Pt layer is also shown.


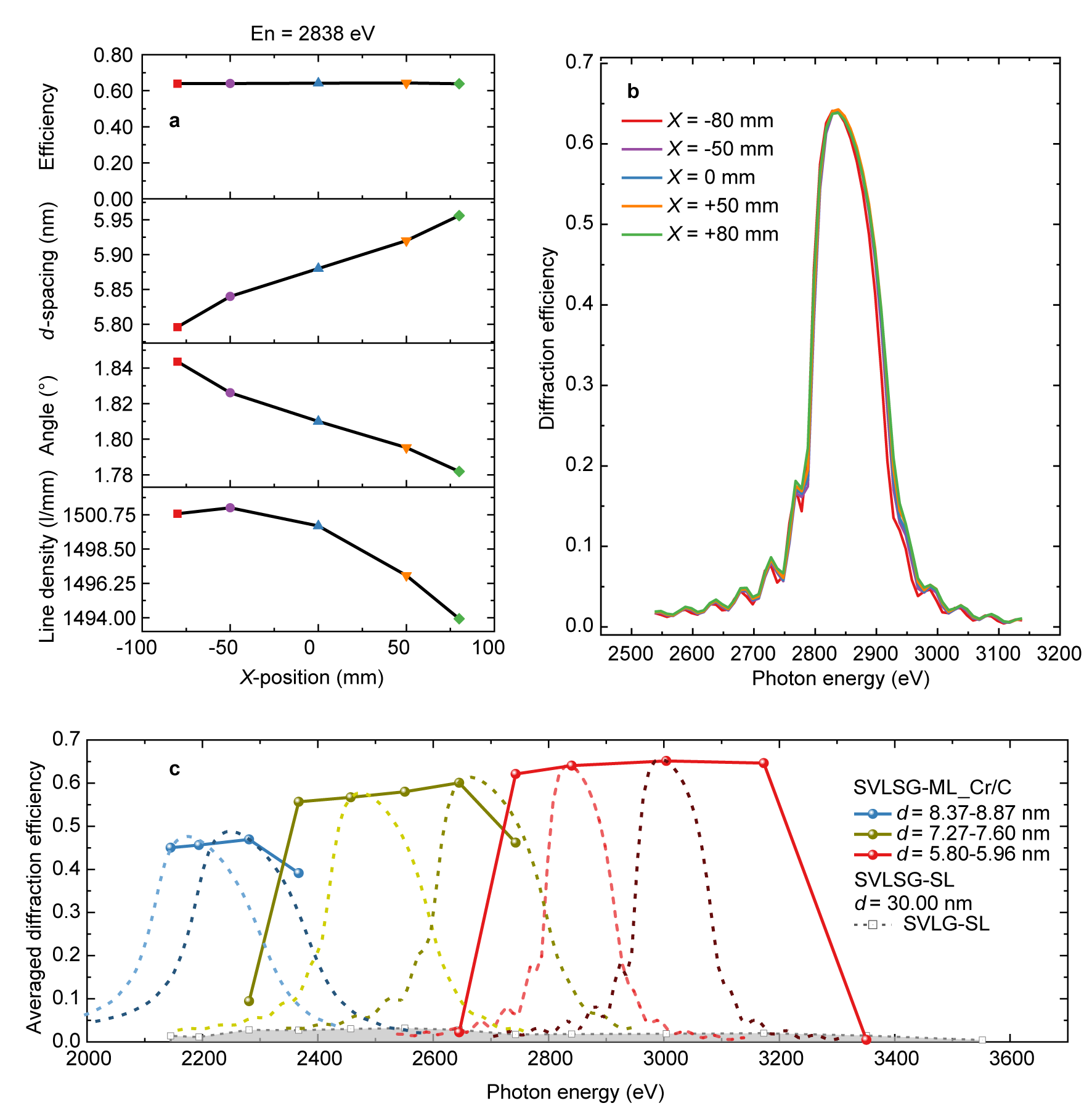


Fig. S1 Design and theoretical performance of the SVLSG-ML. (a) Diffraction efficiency, optimized period thickness, incident angle, and line density as functions of grating position at 2838 eV (from top to bottom). (b) Spectral response curves at different local positions on the grating. (c) Averaged spectral response curves of the SVLSG-ML over different positions for each energy, together with the SVLSG-SL coated by a 30 nm Pt layer.

**1.2 Comparison of the RIXS spectrometer**

A comparison of the current tender RIXS (TRIXS), the RIXS in beamline ID32 at ESRF (ERIXS), and intermediate X-ray RIXS (IRIXS) performance with their energy resolution, photon flux, energy window, and acquisition mode was shown in Table S1. Note that the photon flux of TRIXS at 2838 eV can be further improved by upgrading the beamline monochromator into a multilayer grating type, with the possibility to surpass the current level of IRIXS. The energy resolution can also be improved by using high diffraction order or high line density grating, as mentioned in the main text.

Table S1 Comparison of the performance of RIXS instruments

| **RIXS spectrometer** | **DLS I21** | | **ESRF ID32**  **ERIXS [3]** | **PETRAIII P01 IRIXS [4]** |
| --- | --- | --- | --- | --- |
|  | **Core energy [1]** | **TRIXS [2]** |  |  |
| Energy range | 280-1600 eV | 2000-3000 eV | 400-1600 eV | 2400-4000 eV |
| Coverage | Continuous | Continuous | Continuous | Discrete |
| Energy resolution | 35 meV  @ 930 eV | 334 meV  @ 2838 eV | 32 meV  @ 930 eV | 100 meV  @ 2838 eV |
| Photon flux | 2×10^12^ phs s^-1^  @ 930 eV | 2×10^11^ phs s^-1^  @ 2838 eV | 2×10^11^ phs s^-1^  @ 930 eV | 1×10^12^ phs s^-1^  @ 2838 eV |
| RIXS energy window | ~10 eV  @ 930 eV | ~40 eV  @ 2838 eV | ~10 eV  @ 930 eV | ~1 eV  @ 2838 eV |
| Acquisition mode | Single exposure | Single exposure | Single exposure | Scanning |

**References**

[1] Zhou, K. J. et al*.* I21: an advanced high-resolution resonant inelastic X-ray scattering beamline at Diamond Light Source. *Journal of Synchrotron Radiation* **29**, 563–580 (2022).

[2] Current work.

[3] Brookes, N. B. et al. The beamline ID32 at the ESRF for soft X-ray high energy resolution resonant inelastic X-ray scattering and polarisation dependent X-ray absorption spectroscopy. *Nuclear Instruments and Methods in Physics Research Section A: Accelerators, Spectrometers, Detectors and Associated Equipment* **903**, 175–192 (2018).

[4] Gretarsson, H. et al*.* IRIXS: a resonant inelastic X-ray scattering instrument dedicated to X-rays in the intermediate energy range. *Journal of Synchrotron Radiation* **27**, 538–544 (2020).
